# Prediction of prostate cancer recurrence using quantitative phase imaging: Validation on a general population

**DOI:** 10.1038/srep33818

**Published:** 2016-09-23

**Authors:** Shamira Sridharan, Virgilia Macias, Krishnarao Tangella, Jonathan Melamed, Emily Dube, Max Xiangtian Kong, André Kajdacsy-Balla, Gabriel Popescu

**Affiliations:** 1 Quantitative Light Imaging Laboratory, Department of Bioengineering, Beckman Institute of Advanced Science and Technology, University of Illinois at Urbana-Champaign, 405N. Matthews Avenue, Urbana, IL 61801, USA; 2 Department of Pathology, University of Illinois at Chicago, 840S. Wood Street, Chicago, IL 60612, USA; 3 Department of Pathology, Christie Clinic, University of Illinois at Urbana-Champaign, 1400W. Park Street, Urbana, IL 61801, USA; 4 Department of Pathology, New York University Langone Medical Center, 462 First Avenue, New York, NY 10016, USA; 5 Quantitative Light Imaging Laboratory, Department of Electrical and Computer Engineering, Beckman Institute of Advanced Science and Technology, University of Illinois at Urbana-Champaign, 405N. Matthews Avenue, Urbana, IL 61801, USA

## Abstract

Prediction of biochemical recurrence risk of prostate cancer following radical prostatectomy is critical for determining whether the patient would benefit from adjuvant treatments. Various nomograms exist today for identifying individuals at higher risk for recurrence; however, an optimistic under-estimation of recurrence risk is a common problem associated with these methods. We previously showed that anisotropy of light scattering measured using quantitative phase imaging, in the stromal layer adjacent to cancerous glands, is predictive of recurrence. That nested-case controlled study consisted of specimens specifically chosen such that the current prognostic methods fail. Here we report on validating the utility of optical anisotropy for prediction of prostate cancer recurrence in a general population of 192 patients, with 17% probability of recurrence. Our results show that our method can identify recurrent cases with 73% sensitivity and 72% specificity, which is comparable to that of CAPRA-S, a current state of the art method, in the same population. However, our results show that optical anisotropy outperforms CAPRA-S for patients with Gleason grades 7–10. In essence, we demonstrate that anisotropy is a better biomarker for identifying high-risk cases, while Gleason grade is better suited for selecting non-recurrence. Therefore, we propose that anisotropy and current techniques be used together to maximize prediction accuracy.

In 2010, 138,000 men in the USA underwent radical prostatectomy for treatment of prostate cancer^1^. Biochemical recurrence or increase in serum prostate specific antigen (PSA) levels after prostatectomy is an early sign of prostate cancer recurrence. 17–33% of patients who undergo radical prostatectomy as primary form of treatment experience a biochemical recurrence of prostate cancer and 29–34% of individuals in that cohort will have metastatic prostate cancer with bone as the most common site of metastasis^2^,^3^,^4^,^5^,^6^. The 5-year survival rate for metastatic prostate cancer is 25–43%^3^,^7^. Identification of individuals at high risk for biochemical recurrence will enable early adjuvant treatment for those patients, and thus reduce the risk for metastatic disease and prostate cancer-specific mortality.

Various methods based on pre and post-surgical evaluation of prostate tissue and clinical parameters have been developed for prediction of biochemical recurrence and these techniques have been reviewed elsewhere^8^,^9^. The post-prostatectomy biochemical recurrence prediction methods which are most widely reported and validated are the Stephenson nomogram and CAPRA-S which have concordance index values reported between 0.72–0.77^9^,^10^. Optimistic prediction of non-recurrence is a problem that has previously been noted in both methods, despite the high discrimination accuracy^9^,^11^. Additionally, these methods can lead to erroneous results as they rely on PSA levels and Gleason score (GS) reports which are prone to errors from assay sensitivity and inter-observer variability respectively^12^.

Recently, biomarker-based approaches have been developed as both stand-alone predictors of prostate cancer recurrence and in combination with nomogram-based approaches^13^,^14^,^15^,^16^,^17^,^18^,^19^. The combination of those biomarkers with existing nomograms resulted in improved performance, but they are still subject to the vulnerabilities associated with subjective parameters in nomograms. We previously demonstrated the utility of anisotropy of light scattering in the stromal layer adjacent to cancerous glands, measured using quantitative phase imaging (QPI)^20^, as an independent biomarker for prediction of biochemical recurrence^19^. The study was conducted in a nested case control population where recurrent cases were matched with non-recurrent cases based on age, pTNM stage, primary and secondary Gleason grade. We demonstrated that anisotropy had the ability to identify recurrence with 77% sensitivity and 62% specificity, while CAPRA-S showed poor discriminatory ability as multiple CAPRA-S parameters were used as matching criterion.

In this paper, we present the results from an external validation of optical anisotropy as a biomarker for prostate cancer recurrence. The patient population in this study had a recurrence probability of 17% which is aligned closely with recurrence rates reported in general populations. The aim of this work was to identify specific conditions under which anisotropy should be used as a biomarker for recurrence instead of existing prognostic tools. We performed this study by first testing the performance of anisotropy as a recurrence predictor, and then comparing the performance of anisotropy with CAPRA-S across the low, intermediate, and high risk CAPRA-S groups and finally, comparing the performance of anisotropy with CAPRA-S across GS 5–6, 7 (3 + 4), 7 (4 + 3), 8–10. Our results showed that anisotropy has added value over CAPRA-S at GS ≥ 7; and at CAPRA-S ≥ 3 which corresponds to the intermediate and high risk groups.

## Results

### Anisotropy as a predictor of biochemical recurrence

**Figure 1:**
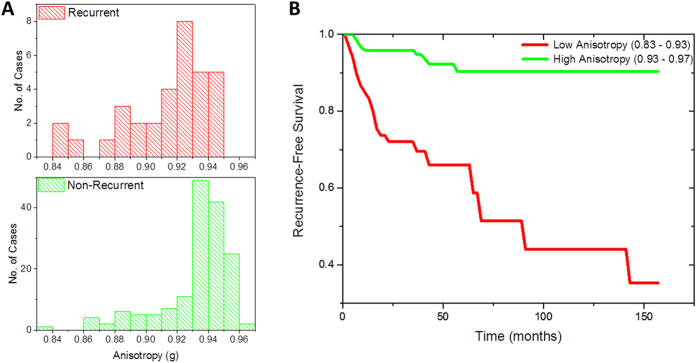
Optical anisotropy as a predictor of prostate cancer recurrence. **(A**) Histograms of the distribution of anisotropy in the single layer of stroma surrounding 6–18 glands from 33 patients with post-prostatectomy biochemical recurrence of prostate cancer and 159 non-recurrent patients. The bin-size on the histogram was set at 0.02. The anisotropy value is lower in the recurrent patients, compared to the non-recurrent patients (One-way ANOVA, p = 7.05 × 10^−5^) **(B)** Kaplan-Meier survival curve with end-point as disease recurrence for 67 patients with low anisotropy values (0.83–0.93) and 125 patients with high anisotropy values (0.93–0.97).

Anisotropy (g) of light scattering was measured using the scattering phase theorem in the unstained prostatectomy samples imaged using spatial light interference microscopy (SLIM), a QPI method (see Materials and Methods for details)^21^,^22^. Anisotropy was calculated in the single stromal layer adjoining 6–18 glands from each of the 33 patients with post-prostatectomy biochemical recurrence of prostate cancer and 159 patients who did not have recurrence. The calibrated anisotropy value in the recurrent cases (0.913 ± 0.028; median = 0.92) was lower than that in the non-recurrent cases (0.932 ± 0.023; median = 0.938) (See Supplementary Information for details). The difference in anisotropy values in the cancer-adjacent stroma from the recurrent and non-recurrent groups was statistically significant (one-way ANOVA, p = 7.05 × 10^−5^). These results are summarized in Fig. 1A. A Kaplan-Meier survival analysis was performed to test the utility of anisotropy for predicting biochemical recurrence as the end-point. The anisotropy ranges tested were 0.68–0.93 (67 patients) and 0.93–0.97 (125 patients) and the results, which are summarized in Fig. 1B, show that patients with low anisotropy values had a higher likelihood of disease progression. The 3-year and 5-year recurrence-free probability dropped from 95% and 90% respectively for patients with high anisotropy values to 70% and 65%, respectively, for patients with low anisotropy values.

### Comparison of Anisotropy, pre-surgical PSA level, Gleason score and CAPRA-S as recurrence predictors

**Figure 2:**
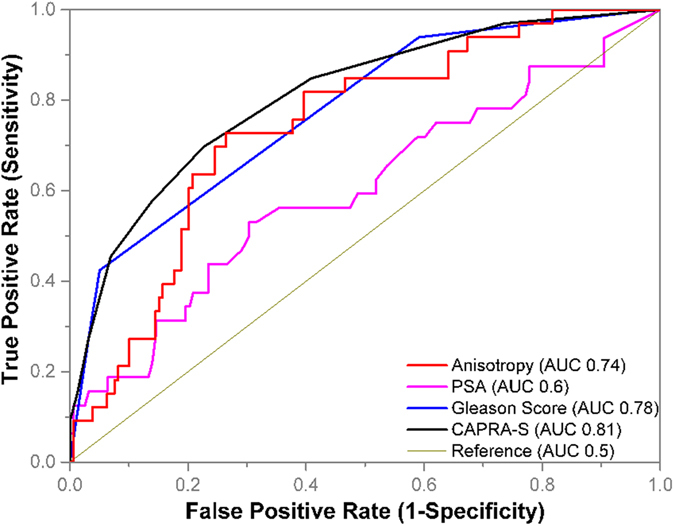
Comparison of recurrence prediction metrics. The performance of anisotropy measured on quantitative phase images, pre-surgical prostate-specific antigen (PSA) levels, Gleason score and CAPRA-S as post-prostatectomy biochemical recurrence predictors was studied in 192 prostatectomy cases (33 recurrent, 159 non-recurrent). The best performance was observed with CAPRA-S (AUC 0.81) and Gleason scores (AUC 0.78). The discriminatory ability of anisotropy (AUC 0.74) was lower than that of CAPRA-S and Gleason score. However, at the optimal performance point, anisotropy had a sensitivity of 72.7% and specificity of 73.6% compared to the 69.6% sensitivity and 77.4% specificity of CAPRA-S. Pre-surgical PSA level (AUC 0.6) was a poor predictor of recurrence.

We compared the ability of anisotropy to distinguish between recurrent and non-recurrent cases using the receiver-operating curve analysis as shown in Fig. 2. Anisotropy had an area under the curve (AUC) of 0.74 (95% CI 0.64–0.84) and at a cut-off value of g = 0.93, recurrence was predicted with a sensitivity of 73% and a specificity of 73%. Pre-surgical PSA, with an AUC of 0.6 (95% CI 0.5–0.7) was not a good predictor of post-prostatectomy biochemical recurrence. In this cohort of patients, representative of a general population, both GS and CAPRA-S out-performed anisotropy as a recurrence predictor. GS with an AUC of 0.78 (95% CI 0.69–0.86) and CAPRA-S with an AUC of 0.81 (95% CI 0.73–0.89) showed comparable performance. At the cut-off value of GS = 6.5, the sensitivity and specificity of recurrence prediction was 93.9% and 41% respectively; and at a cut-off value of Gleason score = 7.5, the sensitivity and specificity of recurrence prediction was 42% and 95% respectively. This steep gradient is reflective of GS 7 prostate cancer being a disease stage with diverse prognosis while GS < 6 and GS > 7 have very low and high risk of recurrence respectively. For CAPRA-S, the optimal cut-off value for recurrence prediction was CAPRA-S = 2.5, resulting in a sensitivity of 69% and specificity of 77%. The optimal performance of CAPRA-S and anisotropy in identifying recurrence in a general population is comparable, with anisotropy being more sensitive and CAPRA-S more specific.

### Anisotropy as predictor of recurrence at various CAPRA-S ranges

**Figure 3:**
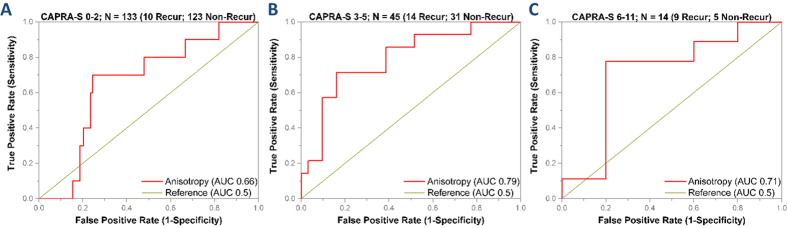
Performance of Anisotropy as a recurrence predictor. The performance of anisotropy as a recurrence predictor was compared across the low (0–2), intermediate (3–5) and high (6–11) CAPRA-S ranges. (**A**) At the low CAPRA-S range, the probability of recurrence was 7.5% and anisotropy (AUC 0.66) predicted recurrence with 70% sensitivity and 75% specificity. (**B**) At the intermediate CAPRA-S range, the probability of recurrence was 31.1%. Anisotropy (AUC 0.79) identified recurrence with 71% sensitivity and 65% specificity. (**C**) At the high CAPRA-S range, the probability of recurrence was 64.3% and anisotropy (AUC 0.71) identified recurrence with 78% sensitivity and 80% specificity.

CAPRA-S has high specificity in recurrence prediction and anisotropy has higher sensitivity. So we compared the performance of anisotropy as a recurrence predictor at the low, intermediate and high risk CAPRA-S ranges as defined previously^11^. At low CAPRA-S risk range of 0–2 CAPRA-S points, the probability of recurrence was 7.5% in 133 patients in the study. Anisotropy could identify recurrence with 70% sensitivity and 75% specificity (AUC 0.66, Fig. 3A). At intermediate CAPRA-S risk range of 3–5 CAPRA-S points, the probability of recurrence was 31.1% in 45 patients. Using anisotropy, recurrent patients could be identified with 71% sensitivity and 65% specificity (AUC 0.79) (Fig. 3B). 14 patients in our study had high risk CAPRA-S scores of 6–11 and the probability of recurrence was 64.3%. Anisotropy could identify recurrence in this group with 78% sensitivity and 80% specificity (AUC 0.71) (Fig. 3C).

### Effect of Gleason score on anisotropy and CAPRA-S as recurrence predictors

**Figure 4:**
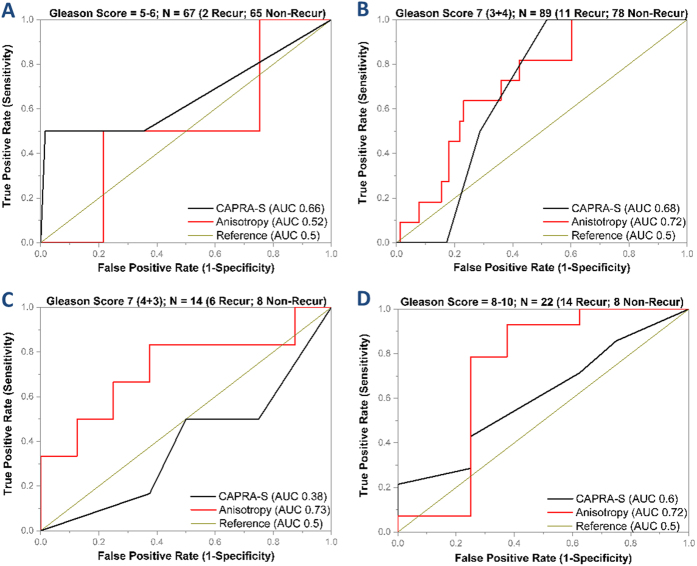
Gleason-score adjusted performance of Anisotropy and CAPRA-S. **(A)** At Gleason scores of 5–6, both anisotropy and CAPRA-S show poor performance at identification of recurrent individuals. However, the probability of recurrence at this stage is 3% and CAPRA-S outperforms anisotropy due to it’s ability to identify 100% of the non-recurrent cases. **(B)** At Gleason score of 7 (3 + 4), anisotropy (AUC 0.72) and CAPRA-S (AUC 0.68) show comparable performance. The probability of recurrence at this stage was 16%. **(C)** At Gleason score of 7 (4 + 3), the probability of recurrence is 43%. CAPRA-S (AUC 0.38) failed due to overestimation of the recurrence risk. Anisotropy was able to identify recurrence (AUC 0.73). **(D)** At Gleason score of 8–10, the probability of recurrence is 64%. Anisotropy (AUC 0.73) was able to identify the recurrent cases with a higher level of accuracy than CAPRA-S (AUC 0.6).

Gleason score is an important component of CAPRA-S, with 0–3 points out of the total possible 0–11 CAPRA-S points sourced from the Gleason grades. The observation that the overall performance of CAPRA-S and GS in our cohort was comparable, led us to study the performance of anisotropy and CAPRA-S at various GS ranges to determine added value. These results are summarized in Fig. 4 and Table 1. In Fig. 4, we show the results from the AUC analysis comparing CAPRA-S and anisotropy at the GS ranges 5–6; 7 (3 + 4), 7 (4 + 3) and 8–10.

At GS 5–6, the probability of recurrence was 2.98% among the 67 patients and all cases had CAPRA-S scores in the low and intermediate range (Fig. 4A, Table 1). At this Gleason range, anisotropy over-estimated the probability of recurrence. While only 1.5% and 50% of patients with low and intermediate CAPRA-S scores, respectively, had a recurrence, CAPRA-S did not provide added value over GS. At GS 7 (3 + 4), anisotropy (AUC 0.72) had better discrimination ability than CAPRA-S (AUC 0.68) (Fig. 4B). For the 89 patients in our study with GS 7 (3 + 4), 70.8% patients had low CAPRA-S scores, 27% had intermediate CAPRA-S scores and 2.2% patients had high CAPRA-S scores; 29.2% had low anisotropy values and 70.8% had high anisotropy values (Table 1). While both patients with high CAPRA-S scores had a recurrence, 9.5% patients with low CAPRA-S and 12.5% with intermediate CAPRA-S scores also experienced recurrence. The probability of recurrence among patients with low and high anisotropy values was 26.9% and 6.3% respectively. Anisotropy, thus had a 63.6% sensitivity and 76% specificity for identifying recurrence at GS 7 (3 + 4) and demonstrated added value over both GS and CAPRA-S.

For the 14 patients with GS 7 (4 + 3), the probability of recurrence was 42.85%. 57.1% patients had low anisotropy values and 42.9% patients had high anisotropy values, whereas, 35.7% patients were classified into the low and intermediate risk categories of CAPRA-S and 28.6% patients were classified into the high risk category of CAPRA-S (Table 1). However, the CAPRA-S grouping and recurrence rates showed an inverse relationship, and therefore CAPRA-S showed poor discrimination in Gleason 7 (4 + 3) patients (AUC 0.38) (Fig. 4C). 62.5% of patients with low anisotropy values had recurrence and 83.3% of patients with high anisotropy did not have a biochemical recurrence. Anisotropy shows the ability to predict recurrence with 83.3% sensitivity and 62.5% specificity (AUC 0.73) and thus outperforms CAPRA-S in the patients with GS 7 (4 + 3).

Our study had 22 patients with GS 8–10 and the probability of recurrence in this cohort was 63.3%. 72.7% of the patients had low anisotropy values and 27.3% of the patients had high anisotropy values (Table 1). There were no patients in the CAPRA-S low risk category since GS 8–10 corresponds to 3 CAPRA-S points^9^. 63.6% and 36.4% patients were in the CAPRA-S intermediate and high risk categories. While the probability of recurrence linearly scales within the CAPRA-S ranges from 57.1% to 75%, the sensitivity of recurrence prediction was low (AUC 0.6, Fig. 4D). 81.3% of the patients with low anisotropy values had recurrence and 83.3% of the patients with high anisotropy values did not experience a biochemical recurrence. The overall sensitivity and specificity of anisotropy as a recurrence predictor in this cohort was 92.8% and 62.5% respectively (AUC 0.73) and thus shows better discrimination than CAPRA-S.

## Discussion

Our results show that a lower value of optical anisotropy in the stromal layer immediately adjoining cancerous glands, which is indicative of a more fractionated stromal morphology, is a strong predictor of prostate cancer recurrence, and therefore poor prognosis. Interestingly, these predictive changes in stromal morphology are more pronounced at advanced stages of disease progression, that is, GS ≥ 7, as opposed to GS 5–6. This suggests that stromal disorganization is a critical requirement for disease progression at advanced disease states. The probability of post-prostatectomy disease recurrence is small at GS 5–6. At this Gleason range, very high sensitivity combined with very high specificity is necessary for identifying recurrence. CAPRA-S has high accuracy at predicting non-recurrence at this disease stage, and therefore outperforms anisotropy.

However, at GS 7, the probability of recurrence varies based on the primary and secondary pattern. The 5-year recurrence rate for Gleason 3 + 4 and Gleason 4 + 3 has been reported to be 14.6–29% and 33.3–38.9% respectively^23^,^24^,^25^,^26^. It is very important to have a prognostic method with high sensitivity at this Gleason range to prevent under-treatment. Anisotropy is a strong recurrence predictor in this cohort as evidenced by both our previous study and the current validation study. At GS 8–10, the probability of recurrence is 63.6% and the heavy reliability of current tools on the Gleason score predispose them to over-estimation of recurrence risk. Anisotropy has both high sensitivity and specificity for recurrence prediction at GS 8–10 and thus shows potential to reduce over-treatment.

A simple explanation for the discrepancy in recurrence prediction accuracy between anisotropy and CAPRA-S stems from the manner in which both tools were originally designed and constructed. Anisotropy as a biomarker was selected using tissue from patients primarily with GS ≥ 7 (81.77%). CAPRA-S was constructed from the CaPSURE database with > 50% of the patients having GS ≤ 6^9^,^11^. Anisotropy was thus designed to work on intermediate and high risk cases, while CAPRA-S was designed for a general population.

Based on our results, we conclude that anisotropy has to potential to improve treatment decisions for patients diagnosed with prostate cancer of Gleason grades 7–10. There is value in the use of prognostic markers post-radical prostatectomy, as evidenced by Decipher^®^, a genomic test offered by GenomeDX that outperforms PSA doubling time as a predictor of metastasis. Similar to optical anisotropy, it finds patients who have good prognosis despite adverse pathology and is approved by Medicare for use in post-prostatectomy patients^27^. However, prognostic marker for use in pre-prostatectomy biopsies is the ultimate goal of our line of studies. We demonstrate here that we can recognize aggressive cancers with this method and the next step will be to use it for biopsies. Since our technique relies solely on parameters measured in prostate tissue, anisotropy can potentially be used as a prognostic biomarker, in conjunction with Gleason scores, for patients undergoing prostate biopsies.

## Materials and Methods

### Prostate Tissue Specimen

We obtained the 217 case biochemical recurrence tissue microarray (TMA) set from the Prostate Cancer Biorepository Network (PCBN). The TMA set has prostatectomy tissue and clinical data associated with patients who were treated for prostate cancer with radical prostatectomy at New York University and informed consent was obtained from all patients. The studies have been performed in accordance with the protocols approved by the Institutional Review Board at the University of Illinois at Urbana-Champaign (IRB Protocol Number: 13900).

### TMA Cohort

The TMA set had prostatectomy tissue from 217 patients with 23 cases of adjacent normal tissue and 13 cases of benign prostatic hyperplasia (BPH). 38 patients had a biochemical recurrence of prostate cancer and 164 patients did not have a biochemical recurrence. There were 4–5 cores per patient and the cores were arrayed across 5 blocks. Biochemical recurrence was defined as a singular PSA reading of ≥ 0.4 ng/ml or PSA level of ≥ 0.2 ng/ml followed by increasing PSA values in subsequent follow-up^28^. Gleason grading was performed by consensus between two uropathologists in this study in accordance with the post-2005 International Society of Urological Pathology (ISUP) definitions for Gleason grading. 192 cases were analyzed in this study and information regarding the patients’ clinical parameters is shown in Table 2.

TMA tissue sections were cut from each of the 5 blocks at New York University. The sectioning thickness was 4μm, which is standard pathology practice. The tissue was de-paraffinized and the unstained tissue slide was cover-slipped with aqueous mounting medium at the pathology laboratory at the University of Illinois at Chicago. The slides were then imaged at the Quantitative Light Imaging Laboratory in Urbana, Illinois using the spatial light interference microscope (SLIM), which is a QPI technique described below.

### Spatial Light Interference Microscopy

The Spatial Light Interference Microscopy (SLIM) system is an add-on module to a phase contrast microscope and is reviewed in detail elsewhere^21^. We used the commercial SLIM system (Cell Vista SLIM Pro, Phi Optics, Inc.) combined with imaging software developed in house, which synchronizes spatial light modulator (SLM) switching, stage scanning and image acquisition. The TMA slides were imaged using the 40 X/0.75NA objective and the size of each field of view, which can be controlled, was set at 85.16 × 82.6 μm^2^. We used a 10% overlap setting on all sides of each frame during mosaic acquisition.

### Sample Analysis

**Figure 5:**
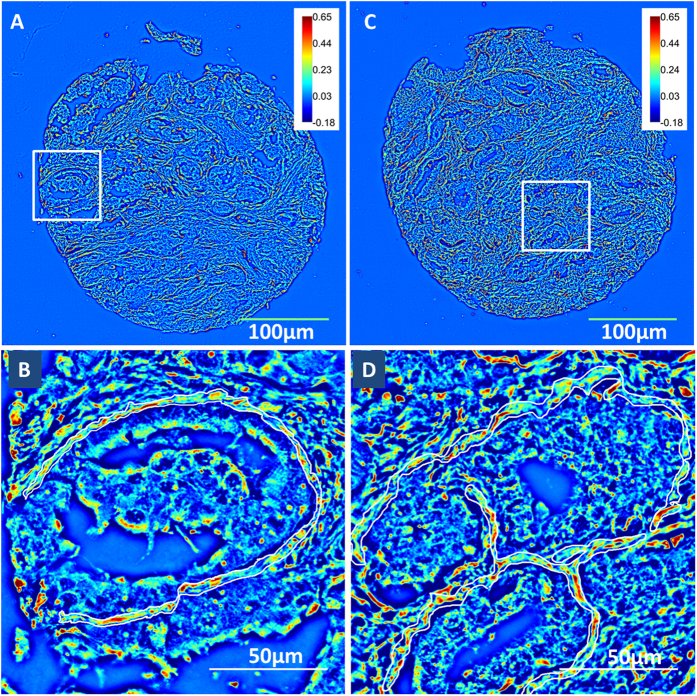
Quantitative Phase Images obtained using Spatial Light Interference Microscopy (SLIM). (**A**) Unstained prostatectomy core from a patient with biochemical recurrence of prostate cancer 3 months after prostatectomy **(B)** Zoomed-in region from the recurrent patient with the single layer of stroma surrounding the Grade 3 cancerous gland marked. Anisotropy in this selected region is computed for recurrence risk calculation. A lower value of anisotropy, or higher degree of fragmentation in the stroma, is observed in the patients at high risk for recurrence **(C)** Unstained prostatectomy core from a patient without biochemical recurrence of prostate cancer for 44 months PSA follow-up time after radical prostatectomy **(D**) Single layer of stroma surrounding a Grade 3 cancerous gland from the non-recurrent patient is marked, and anisotropy was calculated in this selected region. The stromal layer was less fragmented and hence had a higher value of anisotropy.

The Grid and Collection plugin in Fiji was used to stitch the mosaic together. The diameter of each tissue core was approximately 0.7 mm, so the cropped dimensions of the image of each tissue core were set at 5,000 × 5,000 pixels. Fiji was used to perform analysis of the images. Using a Wacom tablet and the region of interest (ROI) feature on Fiji, a single layer of stroma adjoining 5–6 glands from each core was segmented for analysis (Fig. 5). In our study, one core from each patient was arrayed in each of the 3 slides analyzed, and thus we had 6–18 stromal regions for each patient with reduced number of regions in cases were cores were missing. The clear visibility of individual stromal fibers in the SLIM images allows us to accurately segment the stromal fiber immediately adjoining the cancerous glands. The size of each stromal region analyzed varied as a result of variation in glandular size and stromal width. We then measured the phase variance and phase gradient in the stromal region, in order to calculate optical anisotropy (g) using the scattering phase theorem.

### Optical Anisotropy (g)

Optical anisotropy (g) is defined as the average cosine of the scattering angle associated with a single scattering event. Anisotropy can be calculated from the quantitative phase images using the scattering phase theorem^22^:


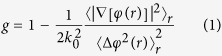


where k_0_ = 2π/λ_0_ is the wavenumber. The center wavelength of the white light used in our imaging system, λ_0_ = 552 nm. Optical anisotropy is calculated over the stromal region, r, corresponding to the ROI. 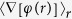 is the mean phase gradient intensity, and 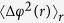 is the phase variance.

Anisotropy value measured is not restricted to the tissue slice imaged and is actually a property of bulk tissue. The phase measurement and therefore g, is representative of the entire tissue when out-of-focus light is negligible. Anisotropy measurements are, therefore, thickness independent (See supplemental for experimental evidence). All samples used in this study were clinical samples that were subject to standard tissue processing methods: formalin fixation and paraffin embedding. Tissue sectioning from the paraffin block and subsequent de-paraffinization, rehydration and slide mounting was performed at the same pathology laboratory for all tissue used in this study. There is no known reason to suspect that prostate tissue would be differentially susceptible to processing artifacts based on the recurrence status of the patient. While standard histology processing techniques can affect tissue architecture, the robustness of anisotropy measurements in the 373 patients from the original study and the validation study leads us to believe that the architectural changes are consistent across all samples.

Precision, and therefore repeatability, is an important requirement for prognostic tools. As in our previous study, we measured the effect of phase noise in a background area (no tissue) and measured its effect on g-values. We repeated this measurement since we used a different imaging configuration in our current study. The error is measured as:


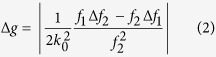


where *f*_1_ and *f*_2_ are the square of the average phase gradient intensity and square of the phase variance, respectively, and are measured over the same background region. Δ*f*_1_ and Δ*f*_2_ are the standard deviations in *f*_1_ and *f*_2_, respectively, due to phase noise. In the current imaging set-up, Δg = 1.52 × 10^−3^. The low error value is attributed to white light imaging, which eliminates speckles, and therefore increases precision.

## Additional Information

**How to cite this article**: Sridharan, S. *et al*. Prediction of prostate cancer recurrence using quantitative phase imaging: Validation on a general population. *Sci. Rep.* **6**, 33818; doi: 10.1038/srep33818 (2016).

## Supplementary Material

Supplementary Information

## Figures and Tables

**Table 1:** Recurrence prediction across Gleason scores.

	Recurrence (No. of Cases)	Anisotropy	Recurrence (No. of Cases)	CAPRA-S	Recurrence (No. of Cases)
Gleason Score 5–6	2.98% (67)	Low	5.2% (19)	0–2	1.5% (65)
				3–5	50% (2)
		High	2.1% (48)	6–11	—
Gleason Score 3 + 4	12.35% (89)	Low	26.9% (26)	0–2	9.5% (63)
				3–5	12.5% (24)
		High	6.3% (63)	6–11	100% (2)
Gleason Score 4 + 3	42.85% (14)	Low	62.5% (8)	0–2	60% (5)
				3–5	40% (5)
		High	16.7% (6)	6–11	25% (4)
Gleason Score 8–10	63.63% (22)	Low	81.25% (16)	0–2	—
				3–5	57.1% (14)
		High	16.7% (6)	6–11	75% (8)

The table shows the percentage of cases with recurrence and the total number of cases in each sub-categories according to stacked Gleason grade and anisotropy or stacked Gleason grade and CAPRA-S score.

**Table 2:** Characteristics of TMA Cohort.

VARIABLE	No. of Cases
No. of Patients	**192**
Recurrent	33
Non-Recurrent	159
Race	
Caucasian	166
African-American	4
Asian	6
Other	8
Unknown	8
Patient Age at Prostatectomy (yrs)	
Median Age (range)	60.5 (41.8–77.2)
Pre-operative PSA (ng/ml)	
Median (range)	5.6 (0.0–29.0)
Pathologic Gleason score	
5–6	67
7 (3 + 4)	89
7 (4 + 3)	14
8–10	22
Extraprostatic Extension	
Positive	3
Negative	189
Seminal Vesicle Invasion	
Positive	14
Negative	178
Surgical Margins	
Positive	33
Negative	159
Mean Follow-up time (months)	
Follow-up Time (range)	59 (2–180)
PSA Follow-up Time (range)	48 (2–175)

This table provides information regarding the total number of patients in the TMA set within each clinical category.
