# Supplementary material for: Prediction of prostate cancer recurrence using quantitative phase imaging: Validation on a general population
Source: Sci Rep. 2016 Sep 23;6:33818. doi: 10.1038/srep33818 (PMC5034339; doi:10.1038/srep33818)
Supplement: Supplementary Information [file srep33818-s1.pdf]

Supplemental Information

Prediction of prostate cancer recurrence using quantitative phase imaging: Validation on a general population

Shamira Sridharan[1], Virgilia Macias[2], Krishnarao Tangella[3], Jonathan Melamed[4], Emily Dube [4], Max Xiangtian Kong[4], André Kajdacsy-Balla[2], and Gabriel Popescu[5,]*

1. System Calibration for Anisotropy Measurements

Optical anisotropy, as measured with the *scattering phase theorem*, is a function of phase gradient and phase variance. However, measurement of phase gradient and variance are sensitive to differences in measurement conditions, such as the number of pixels corresponding to each micron, or physical dimensions of the CCD camera, and the angles measured by an optical system. In our previous study, we used a research-grade Spatial Light Interference Microscopy (SLIM 1.0) for measurement of quantitative phase images (QPI), and it had a different optical configuration from Cell Vista SLIM Pro (SLIM Pro), which was used in this study. Additionally, while we used the same magnification objective, 40X/0.75NA on both systems, the physical dimensions of the camera were different on the two systems, with 14pixels per micron on SLIM 1.0 and 6.3 pixels per micron on SLIM Pro.

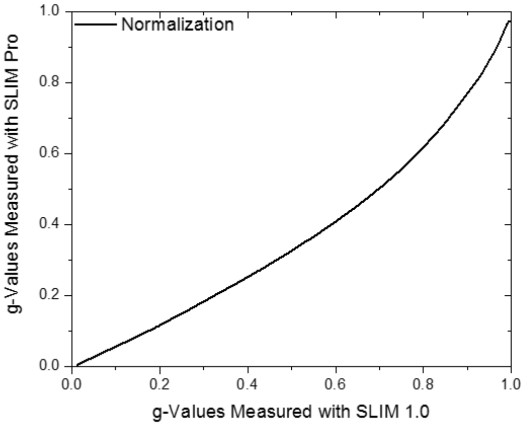

Figure S1: Calibration Curve for Anisotropy. The calibration curve normalizes anisotropy measurements from the SLIM Pro system to the anisotropy measurements obtained using SLIM 1.0. Overall, anisotropy values measured using SLIM 1.0 are higher than those from the SLIM Pro system.

In order to normalize the measurement of anisotropy across the two imaging systems, we used a cumulative distribution function based a transformation approach. A prostatectomy tissue core from the OCTMA5 data-set (Cooperative Prostate Cancer Tissue Resource, Chicago), which was used in our previous study, was re-imaged using the SLIM Pro imaging system. Anisotropy was calculated in the entire tissue core using a 4.42 micron averaging window, corresponding to the average width of individual stromal fibers surrounding cancerous glands. Histograms of anisotropy over a range of 0 to 1 was calculated with 512 bin width on the SLIM 1.0 images and 1024 bin width on the SLIM Pro system. The histograms were converted to cumulative distribution functions and a 1-to-1 anisotropy mapping was performed by matching the height of the histogram corresponding to each g-range. The calibration curve obtained through

this method is shown in Fig S1. The anisotropy values measured using SLIM Pro are lower than the measurements from SLIM 1.0. The SLIM Pro system is more sensitive to finer features, which results in more isotropic scattering and lower g-values.

Tissue preparation is another possible source of variation in anisotropy measurements. In order to determine if systemic calibration is the only source of variation in g-values, we performed a 2-step validation:

1. We measured the anisotropy cut-off value for optimal prediction of biochemical recurrence using the prostatectomy samples from the Prostate Cancer Biorepository Network (PCBN) that was used in this study. The PCBN samples were sectioned at New York University and were de-paraffinized and cover-slipped using aqueous mounting medium at the University of Illinois at Chicago. The un-calibrated cut-off value for recurrence prediction was g = 0.82, which after calibration corresponded to g = 0.93.

2. We imaged prostatectomy samples obtained from CPCTR using both the Pro system and the SLIM 1.0 system. The CPCTR samples were sectioned at the University of Illinois at Chicago, de-paraffinized and cover-slipped using aqueous mounting medium at Presence Covenant Medical Center, Urbana, Illinois.

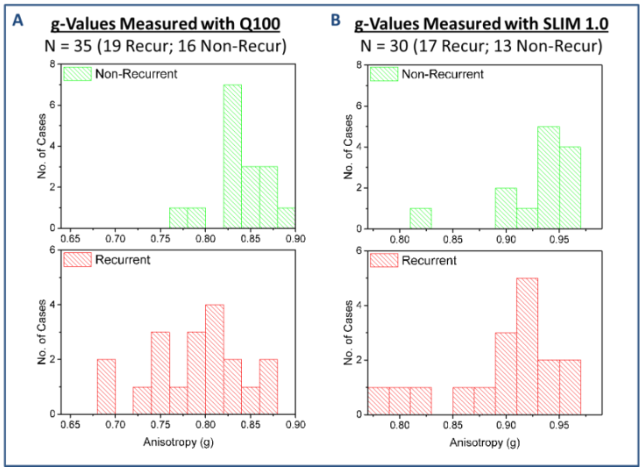

Figure S2: Precision of Anisotropy Measurements. A) In order to measure the precision of the calibration curve, anisotropy was measured on 70 cores from 35 patients in the CPCTR TMA set using Q100 imaging system. The optimal cut-off value for recurrence prediction was determined to be g=0.82, corresponding to the un-calibrated g cut-off value measured for the PCBN TMA set. The calibrated value is g= 0.93. **B)** Anisotropy was measured on 120 cores from 30 patients in the CPCTR TMA set using the SLIM 1.0 imaging system. The optimal cut-off value for recurrence prediction was g=0.93 corresponding to the calibrated value from the Q100 system.

Prostatectomy tissue from 35 patients (19 with recurrence and 16 without recurrence), with 2 cores per patient, were imaged using the SLIM Pro system. Anisotropy measurements were performed on a single layer of stroma surrounding 6-12 cores per patient. The un-calibrated optimal cut-off value for recurrence prediction was g = 0.82 and after calibration, this corresponded to g = 0.93 (Fig S2-A). This corresponded to the cut-off value obtained for the PCBN TMA imaged using the SLIM Pro system.

Prostatectomy tissue from 30 patients, that was imaged using SLIM Pro, was also imaged using the SLIM 1.0 imaging system. Anisotropy measurements were performed on a single layer of stroma surrounding 6-16 glands per patient. The optimal cut-off value for recurrence prediction in that cohort was g = 0.93 (Fig S2-B). This value corresponds to the post-calibration cut-off value from SLIM Pro.

2. Sectioning Effects of Anisotropy Measurements

Anisotropy measurements are independent of thickness, when the light transmitted through tissue is in focus. Here, we show results from an experiment measuring the uniformity of pathology tissue sections in small regions of tissue, corresponding to a tissue core and also study the effect of tissue sections of varying thickness on anisotropy measurements.

Paraffin-embedded thyroid tissue was sectioned at 3.5 μm thickness and placed on a glass slide. The sample was coated with a standard gold/palladium target using a sputter coater (Denton Vacuum, Desk-1 TSC). A cross-section of the metal-coated tissue slide was then imaged using a scanning electron microscope (FEI Company, Phillips XL30 ESEM-FEG), and the images are shown in Fig S3-A, B. A sectioning artifact would manifest itself as a uniform reduction in thickness in a given direction. The reduction in thickness followed by an increase, as seen in two different tissue regions, indicate that the thickness non-uniformities are a result of variations in tissue morphology.

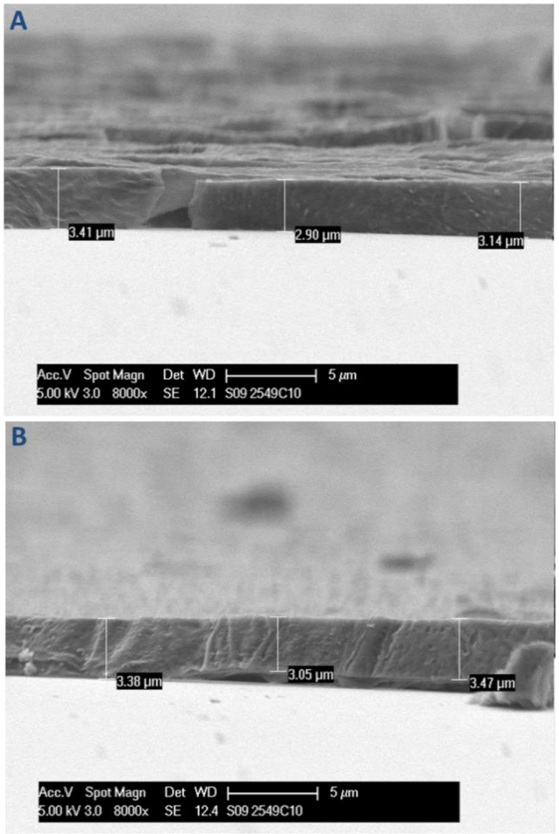

Figure S3: Determination of Tissue Thickness. A) Paraffinized thyroid tissue of 3.5μm sectioning thickness was coated with a standard gold/palladium target and imaged using a scanning electron microscope. The thickness of the tissue shows a small decrease, followed by an increase, indicating changes in tissue morphology. **B)** The same trend is seen in a different tissue region on the same slide.

If the changes in tissue thickness are solely the result of morphology changes, the anisotropy measurement would not vary from one section to the next, in serial sections. To this end, we obtained a tissue microarray slides from the University of Illinois at Chicago, consisting of normal prostate and prostate cancer cores serially sectioned at differing thicknesses over the range of 2μm - 6μm. A 4μm section was cut adjacent to each of the 2, 3, 5 and 6 micron sections as an internal control. Anisotropy was measured in a 32x32μm stromal region adjacent to the same gland in the serial sections of a normal and cancerous core from the microarray. The size of the stromal region corresponded to the stromal regions used in anisotropy analysis for recurrence prediction. Our results show that small variations in thickness (±1μm) do not affect anisotropy values. Additionally, the repeatability of anisotropy measurements in the serial sections shows that thickness non-uniformities over small tissue regions are a property of tissue.

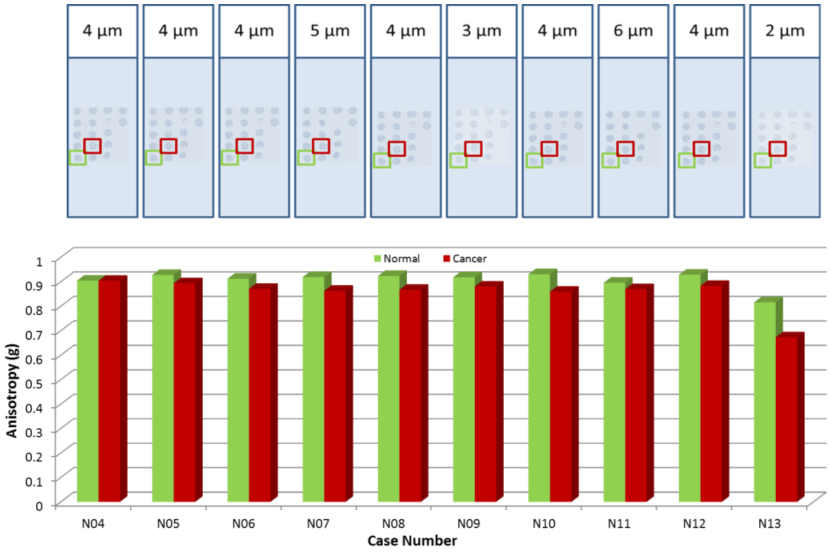

Figure S4: Effect of Section Thickness on Anisotropy. Anisotropy was measured in a 32x32µm stromal region adjoining the same gland from a normal and cancerous core across sections of thicknesses from 2µm – 6µm. The anisotropy measurements are comparable across the different thicknesses, indicating that a local change in tissue thickness is a function of morphology.
